# Strong interactions between learned helplessness and risky decision-making in a rat gambling model

**DOI:** 10.1038/srep37304

**Published:** 2016-11-18

**Authors:** José N. Nobrega, Parisa S. Hedayatmofidi, Daniela S. Lobo

**Affiliations:** 1 Department of Psychiatry, University of Toronto, Toronto, ON, Canada; 2 Departments of Psychology and Pharmacology & Toxicology, University of Toronto, Toronto, ON, Canada; 3 Campbell Family Mental Health Research Institute, Centre for Addiction and Mental Health, Toronto, ON, Canada; 4 Institute of Medical Science, University of Toronto, Toronto, ON, Canada

## Abstract

Risky decision-making is characteristic of depression and of addictive disorders, including pathological gambling. However it is not clear whether a propensity to risky choices predisposes to depressive symptoms or whether the converse is the case. Here we tested the hypothesis that rats showing risky decision-making in a rat gambling task (rGT) would be more prone to depressive-like behaviour in the learned helplessness (LH) model. Results showed that baseline rGT choice behaviour did not predict escape deficits in the LH protocol. In contrast, exposure to the LH protocol resulted in a significant increase in risky rGT choices on retest. Unexpectedly, control rats subjected only to escapable stress in the LH protocol showed a subsequent decrease in riskier rGT choices. Further analyses indicated that the LH protocol affected primarily rats with high baseline levels of risky choices and that among these it had opposite effects in rats exposed to LH-inducing stress compared to rats exposed only to the escape trials. Together these findings suggest that while baseline risky decision making may not predict LH behaviour it interacts strongly with LH conditions in modulating subsequent decision-making behaviour. The suggested possibility that stress controllability may be a key factor should be further investigated.

Risky decision-making is a characteristic of depression and of addictive disorders, including problem gambling^1^,^2^,^3^,^4^,^5^,^6^,^7^,^8^,^9^. However it is not clear whether predisposition towards risky choices increases the likelihood of depressive disorders, whether the converse is the case, or whether the two traits are independently associated to a third factor. One common factor in both addictive and depressive disorders is significant sensitivity to stress; exposure to stress in both humans and animals has been associated with increases in drug seeking and risky decision-making^10^,^11^,^12^. Stress is also strongly implicated in depressive illness, and it is noteworthy that virtually all animal models of depression are stress-based^13^. An increased sensitivity to the depressive impact of stress could potentially increase vulnerability to engage in risky choices, gambling, and other types of addictions. Conversely, traits predisposing to risky or disadvantageous decision-making could conceivably increase vulnerability to depression-inducing effects of stress. The latter possibility has received less experimental attention than the former.

Preclinical models could add valuable insight into the relationships between depressive-type behaviour and risky decision-making, particularly since the direction of such relationships is typically difficult to determine in human populations. Among the better established models of depression is learned helpleness (LH), as a general protocol where exposure to inescapable stress subsequently affects escape responding or ability to cope, presumably by inducing a state of “helplessness”^14^,^15^,^16^. In the last few years behavioural paradigms have also been successfully developed to model gambling and decision-making behaviour in rodents^17^,^18^,^19^. Here, we investigated the effects of learned helplessness on risky decision-making as assessed in the rat Gambling Task (rGT)^18^, a paradigm based on the human Iowa Gambling Task where amount of reward and probability of reward are inversely related. In the rGT behavioural choices associated with high reward/low probability (“high risk, high reward”) are commonly regarded as indices of risky or “impulsive” decision-making, as recently supported by a meta analysis^20^. To address potential bidirectional effects between rGT and LH, our overall experimental approach was to use the rGT to establish a baseline of decision-making behaviour, then expose the animals to a learned helplessness protocol, and then reassess behaviour in the rGT. We were particularly interested in testing the hypothesis that exposure to the LH protocol would increase risky decision-making in the rGT.

## Methods

### Subjects

Male Fischer-344 rats (*n* = 45) (Charles River, Quebec), initially weighing 250–275 g, were individually housed in a temperature-controlled colony room on a 12 h light–dark cycle (lights on at 8:00 A.M.; temperature 21 ± 1 °C). After one week of acclimation with free access to food and water, rats began a food restriction protocol (20–22 g per day) to stay at approximately 85% of their initial free feeding body weight. Water was available *ad libitum* throughout the study. Experimental protocols were approved by the Animal Care Committee at the Centre for Addiction and Mental Health and complied with Canadian Council on Animal Care and NIH standards and guidelines.

### Rat Gambling Task (rGT)

rGT training began one week following the start of food restriction. Animals were tested between 8:00 AM and 3:00 PM five days per week. The rGT experimental protocol has been previously described^18^,^21^. Testing was carried out in commercial chambers (30.5 × 24 × 21 cm; Med Associates Inc., St. Albans, VT, USA) in sound-attenuated cabinets. Each chamber had four active response holes on one wall and a food dispenser on the opposite wall. Each response hole (termed P1, P2, P3, and P4) was associated with the delivery of a specific number of sucrose pellets (1, 2, 3 or 4, respectively) each with a defined probability (0.9, 0.8, 0.5 and 0.4, respectively). Rewards consisted of 45-mg sucrose pellets (Formula P, BioServ, New Jersey). ‘Punishment’ in this paradigm consists of corresponding time-out periods for the non-rewarded trials (5, 10, 30, or 40 sec, respectively), with probabilities of 0.1, 0.2, 0.5 or 0.6, respectively, for each of the 4 choices^18^. A fixed intertrial interval (ITI) of 5 sec was used. Since each session had a fixed duration of 30 min, the choice that maximized the number of possible pellets to be earned in a session was P2, followed by P1, P3 and P4. For each session the choice score for each of the four rGT options was calculated as a percent (number of choices of a particular option/number of total choices made in the session). Also recorded were the number of omissions (failures to make a choice within a trial), premature responses (making a choice before the 5-sec ITI ended), and perseverative responses per session. In the rGT trained rats typically show a very high preference for the optimal P2 choice, usually around 80% of the choices made, with the riskier P3 and P4 choices occurring only 7–10% of the time.

Daily rGT sessions proceeded until rats showed a stable responding over three consecutive sessions, which in this study was achieved after 15 sessions. Since there was a particular interest in risky, disadvantageous decision-making behaviour, we focused on the option associated with maximal possible gain with the lowest probability of occurrence (P4). Mean percent P4 responding across all sessions was therefore used to define three levels of “impulsivity”: High impulsivity (HI) rats were defined as those with a mean P4 response level in the upper 33% of the P4 distribution; rats with mean P4 levels in the middle 33% of the distribution were classified as Medium Impulsivity (MI); and those in the lower 33% of the P4 distribution were classified as Low Impulsivity (LI) animals.

### Learned helplessness protocol and rGT retest

**Figure 1:**
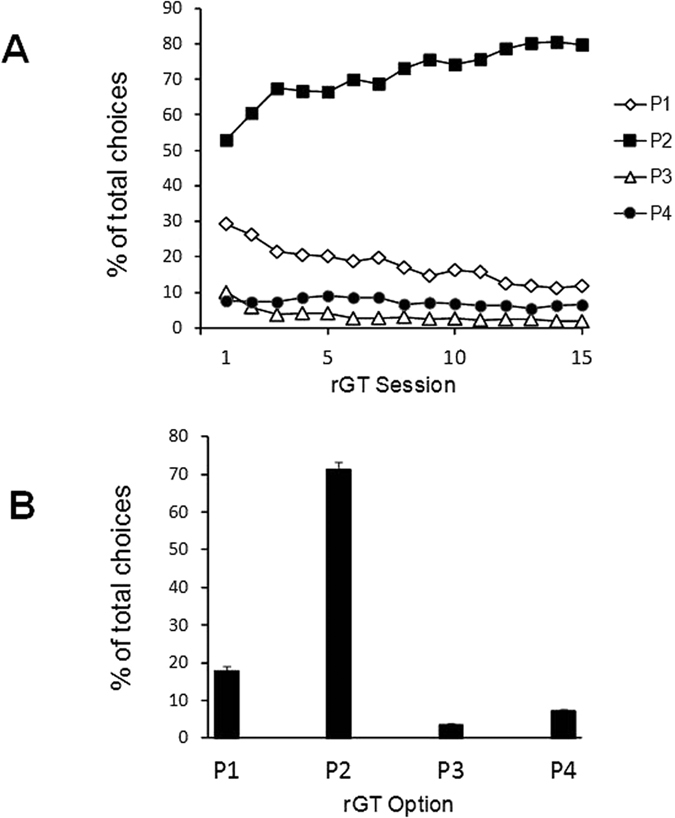
Baseline rGT choice behaviour. (**A**) Distribution of choice behaviour for all rats (N = 45), expressed as mean percent of total choices per session in the course of 15 sessions. Error bars are omitted for clarity. (**B**) Mean and SEM values for each choice averaged over the 15 sessions. On average rats showed a strong preference for P2 option, followed by P1, P4 and P1.

Once stable rGT responding was achieved, a 12-day rest period was given and then rats were subjected to the stress protocol. Forty-five rats were exposed to the LH protocol. Following well established procedures, the target LH group (N = 27) was exposed to one session of inescapable stress (0.8 mA scrambled footshocks) delivered on in operant boxes (35 × 30 × 21.5 cm; Med Associates, St Albans, VT). One hundred shocks of variable duration (1.5–60 sec) were given at variable intervals (1–30 sec) resulting in a total 25 min of shock over the entire session. Twenty-four hours later this group was subjected to an active avoidance session consisting of 15 trials where footshock could be terminated by a single bar press. The maximal duration of each trial was 60 sec and a fixed intertrial interval of 15 sec was used. For each trial latency to escape footshocks was recorded. Impaired escape learning in this group is commonly described as “learned helplessness”^15^,^22^. Impaired escape behaviour in LH protocols is defined by comparison to a separate group that undertakes the active avoidance test without prior exposure to inescapable stress (e.g. refs 23, 24, 25, 26). This group is referred to as the Learning Control (LC) group, as it serves to verify that rats not previously stressed can in fact learn the avoidance task (N = 10). A third group (N = 8) was not exposed to avoidance trials and remained in their home cages (Cage Control groups, CC). The pre-stress baseline distribution of rGT choices was the same for the three groups (LH, LC and CC) in the LH protocol (Supplymentary Fig. 1). The target LH group included 10 LI, 10 HI and 5 MI rats. An attempt was made to distribute the remaining LI, MI and HI rats equally among CC and LC groups (Table 1).

Once the LH protocol was completed all rats were given a 10-day rest period and were then reassessed in the rGT protocol (7 sessions), as described above.

### Statistical analyses

Statistical analyses were conducted with SPSS (version 21, Chicago, IL, USA). Pre- vs. post LH protocol changes were analyzed with MANOVA or repeated measures ANOVA using test time (pre- vs. post-LH protocol) as a within-subjects factor and LH group (LH, LC or CC) and/or Impulsivity (HI, MI or LI) as between-subject factors. Post-hoc tests used Bonferroni-adjusted comparisons, with *p* < 0.05 as a criterion for statistical significance.

## Results

### Baseline rGT performance

**Figure 2:**
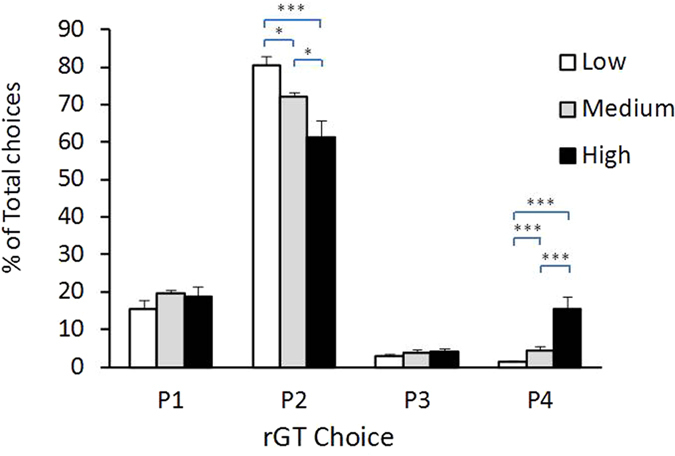
Baseline rGT choice distribution of Low, Medium and High Impulsivity rats. Pre-stress levels of rGT for the three Impulsivity groups, defined as the lower, medium and higher 33% of the P4 distribution. Accordingly, N = 15 for each group. As intended, High-impulsivity rats had higher P4 values and lower P2 values than the other groups. *p < 0.05; **p < 0.01, Bonferroni-adjusted comparisons.

Choice distribution over the sessions is shown in Fig. 1a. As summarized in Fig. 1B, rats on average significantly favoured the optimal P2 option (71.4% ± 0.078 of all choices made per session), followed by P1 (17.9% ± 0.05), P4 (7.2% ± 0.01) and P3 (3.5% ± 0.02). This choice pattern was established relatively early and appeared quite stable over time. When animals were divided into High-, Medium- or Low-impulsivity subgroups on the basis of their mean P4% scores (Fig. 2), one-way ANOVAS confirmed that the three groups differed significantly in P2 and P4 choices (F_2,42_  = 8.04, *p*  < 0.001 and F_2,42_ = *p* < 0.0001, respectively), as intended. Bonferroni-adjusted post-hoc tests confirmed that HI rats significantly favoured P4 significantly more often that LI and MI rats (*p* < 0.05) while showing a corresponding decrease in preference for P2 (*p* < 0.05). The three Impulsivity groups did not differ among themselves in P1 or P3 choices, nor in number of omissions or premature responses (all *p* values >0.05).

### Active avoidance in the LH protocol

**Figure 3:**
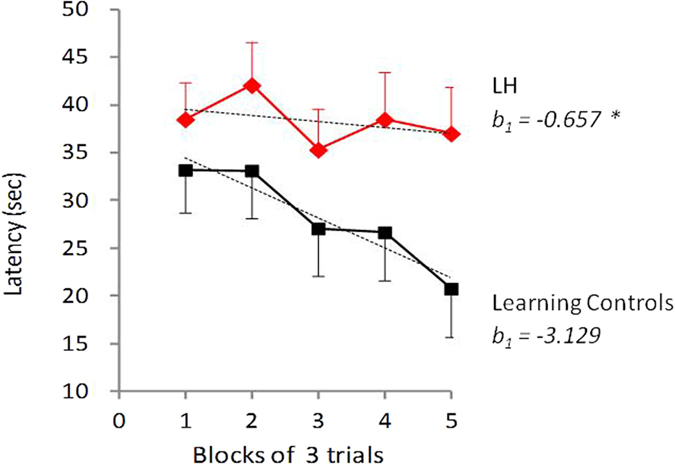
Escape latencies across 15 active avoidance trials. LH rats (N = 27) had one prior session of inescapable stress, whereas LC (N = 10) only experienced the escapable stress session. Although the LH group X Trials ANOVA interaction was not statistically significant, the slopes of the two regression lines were significantly different *p < 0.05. Values are mean latencies to escape footshock (and SEM) in blocks of 3 trials.

Figure 3 shows latencies to escape for LH and LC groups over the 15 trials of the active avoidance session. While latencies in the LC group declined sharply over the 15 trials, latencies in the LH group seemed to decline at a slower rate if at all (Fig. 3). Although a repeated measures ANOVA did not indicate a significant Trials X LH group interaction (F_4,124_ = 0.95, *p* = 0.44), statistical comparison of the two linear slopes indicated a significant difference between the two groups (t_8_ = 2.44, p < 0.04).

### Effects of baseline “impulsivity” on LH behaviour

To examine potential relationships between baseline impulsivity and escape deficits in the LH paradigm a continuous “impulsivity score” was created by computing a ratio of the two riskiest rGT choices (P4 and P3) against the two safest choices (P2 and P1). This impulsivity score was then used to assess Pearson’s correlations between impulsivity and latency to escape (Table 2), first for all tested rats, and then for LH and LC groups separately. As shown in Table 2, no significant correlations were found, suggesting that baseline impulsivity does not predict escape deficits in the LH paradigm. Nor were any significant correlations observed when latencies were broken down into categories (e.g. less than 5 sec; between 5 and 20 sec; and over 20 sec) (Table 2). Similar correlation analyses performed for premature responding in the rGT likewise failed to indicate any association between premature responding at baseline and subsequent escape response in the LHs protocol (Table 2).

### rGT choice behaviour after the LH protocol

**Figure 4:**
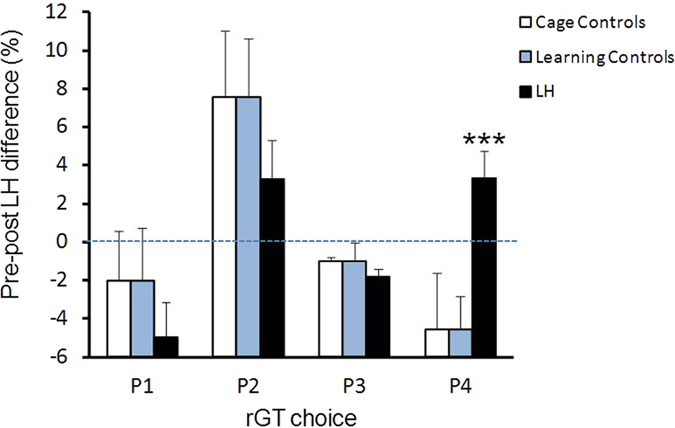
Pre-post LH protocol changes in rGT choices in the 3 LH conditions. After the LH protocol, a general tendency was observed for CC (N = 8) and LC (N = 10) rats to decrease P1, P3 and P4 responding and to increase P2 responding. In contrast rats in the LH stress group (N = 27) showed a significant increase in P4 choices relative to their own pre-stress baseline (***p < 0.0001, paired t test). Values are mean difference (SEM) scores; the dotted line indicates the 0% change value.

The pre-LH baseline distribution of rGT choice behaviour was the same for each of the three groups in the LH protocol (Supplymentary Fig. 1). In order to assess the effects of the LH protocol on rGT retest behaviour repeated measures ANOVAs were performed for each rGT choice, using LH protocol group as a between-subject factor with three levels (LH, LC and CC) and Time (pre- and post-LH) as a within-subject factor. Results indicated a significant main effect of Time for P1 (*F*_*1*,*42*_ = 10.16, *p* = 0.003), P2 (*F*_*1*,*42*_ = 12.77, *p* < 0.001) and P3 choices (*F*_*1*,*42*_ = 18.53, *p* = 0.0001). No significant main effects of the LH protocol were observed. A significant interaction between Time × LH was only seen for the P4 choice (*F*_*2*,*42*_ = 4.43, *p* = 0.018). This suggested that the three conditions in the LH phase of the study differentially affected subsequent P4 selection in the rGT. Bonferroni-adjusted pairwise comparisons revealed that rats in the LH group significantly increased subsequent P4 selection compared to rats in the LC group (*p* < 0.005). Figure 4 depicts this result using pre- vs. post-LH differences.

**Figure 5:**
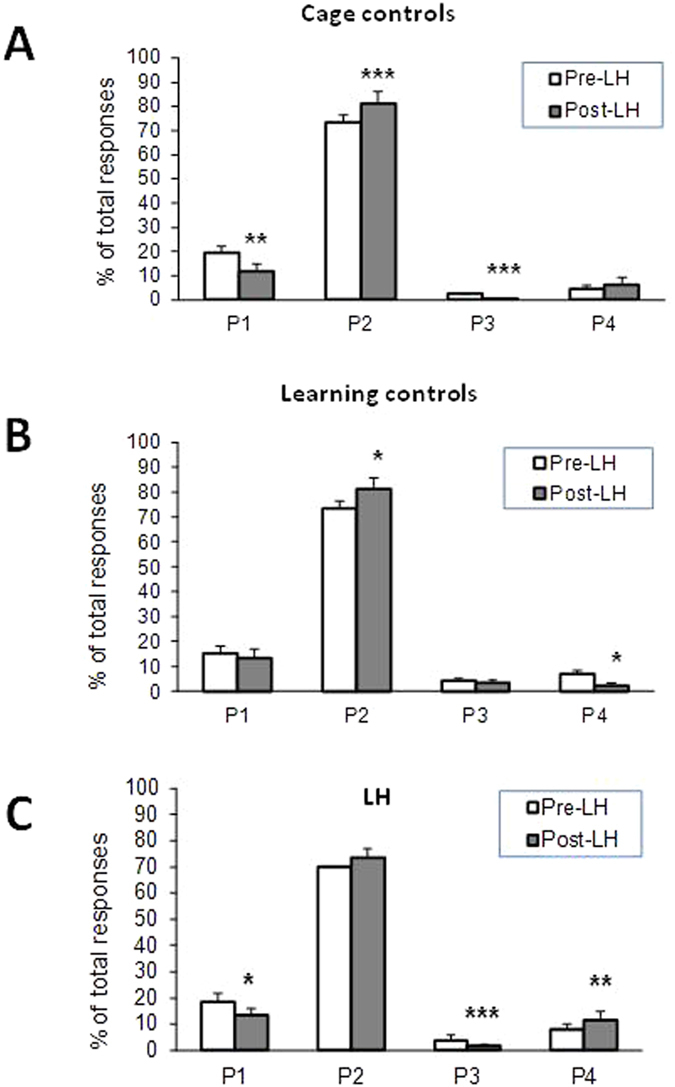
rGT choice changes in Cage Control, Learning Control and LH groups. (**A**) CC rats (N = 8) showed a significant increase in P2 (*p* < 0.01) and a significant decrease in P1 (*p* < 0.02) on rGT retest. (**B**) LC rats (N = 10) subjected to a single active avoidance session showed a significant increase in P2 (*p* < 0.05) and a significant decrease in P4 (*p* < 0.05) on rGT retest. (**C**) LH rats (N = 27) showed a significant increase in P4 responding (*p* < 0.025) and significant decreases in P1 (*p* < 0.05) and P3 (p < 0.01) on rGT retest. Values are means and SEMS. *p  < 0.05, **p < 0.025, ***p < 0.01 pre- vs. post-stress comparisons.

Pre- vs. post-LH choices in the rGT were then analyzed for each group in the LH protocol, using a multivariate ANOVA (MANOVA) that considered the four rGT choices simultaneously. In the CC group (Fig. 5A) significant pre- vs. post-LH protocol differences were seen for P1 (*F*_*1*,*7*_ = 8.470, *p* = 0.023), P2 (*F*_*1*,*7*_ = 5.18, *p* = 0.023) and P3 (*F*_*1*,*7*_ = 106.15, *p* = 0.0001). The slight increase in P2 in this group thus may have been due primarily to a decrease in P1 and P3 (Fig. 5A), consistent with possible effects of additional rGT trials in the absence of other manipulations. In the LC group (Fig. 5B), a significant pre- vs. post-LH difference was seen for P2 (*F*_*1*,*9*_ = 6.31, *p* = 0.033) and P4 (*F*_*1*,*9*_ = 6.80, *p* = 0.028). Figure 5B suggests that the decrease in P4 derived from an increase in P2. In the target LH group (Fig. 5C), significant pre- vs. post-LH effects were seen in P1 (*F*_*1*,*26*_ = 7.39, *p* = 0.012), P3 (*F*_*1*,*26*_ = 22.14, *p* = 0.0001) and P4 (*F*_*1*,*26*_ = 5.54, *p* = 0.026). In this group increases in P4 may have been due to decreases in P1 and P3. In all cases the data in Fig. 5 indicate that the optimal P2 responses remained the overwhelming choice in all groups.

### Effects of the LH protocol on P4 selection in High- vs. Low-Impulsivity rats

**Figure 6:**
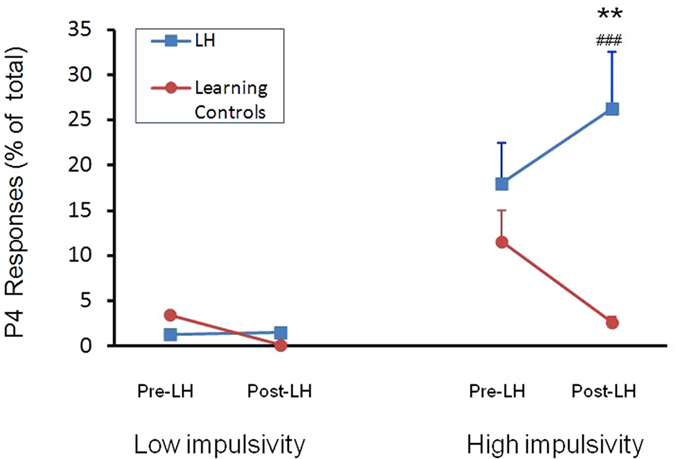
Effects of the LH protocol on Low vs. High Impulsivity rats. Rats defined as a low impulsivity at baseline (N = 15) did not show significant pre-post LH differences. In contrast, rats showing high impulsivity at baseline (N = 15) reacted in opposite ways when exposed to LH vs. Learning Control conditions. Values are means and SEMS. ^###^*p* < 0.01 compared to their own pre-stress values; **p < 0.025 compared to the post-stress Learning Control group.

The data in Fig. 5B and C suggested that the LH protocol could be having opposite effects on P4 responding in rats in the LH group as compared to LC rats. To further explore this possibility the two extreme Impulsivity groups (High and Low) and the two groups subjected to avoidance sessions (LH and LC) were subjected to further analyses focusing on the target “high risk, high reward” P4 response. ANOVA revealed a significant 3-way interaction between Time × LH group ×  Impulsivity (*F*_*1*,*28*_ = 7.48, *p* = 0.011), a significant interaction of LH group × Impulsivity (*F*_*1*,*28*_ = 11.97 *p* = 0.002), and significant main effects of Impulsivity (*F*_*1*,*28*_ = 20.63, *p* < 0.0001) and LH group (*F*_*1*,*28*_ = 11.00, *p* = 0.003) on P4 choice. As can be seen in Fig. 6, following the LH protocol Low-impulsivity rats did not show much change in P4 selection compared to their own pre-LH levels, and this was true for both LH and LC rats (Fig. 6). In contrast, High-impulsivity rats in the LH group showed a significant increase in P4 choices as compared both to their own pre-LH levels (paired *t*_*6*_ = 3.97, p = 0.007) and to the LC group (independent *t*_*8*_ = 2.78, p = 0.024). While high-impulsivity rats in the LC condition decreased their P4 responding by approximately 30% after the stress protocol, High-impulsivity animals in the LH group showed a 56% increase in P4 responding following exposure to stress (Fig. 6).

### Effects of the LH protocol on subsequent omissions, premature responding and perseverative responses in the rGT

Following the same analysis strategy as used for rGT choice options, omissions were found to be unaffected by the LH protocol (Supplymentary Fig. 2). A significant reduction in the number of premature responses was observed in CC rats (−35%, *p* < 0.05) but not in LC or LH rats. When perseverative responding was considered, significant and uniform reductions were seen in all three groups in the LH protocol (39–40%, all *p* values <0.01; Supplymentary Fig. 2).

## Discussion

Three main findings emerge from this study. First, baseline impulsivity, as defined by risky choices in the rGT, did not predict escape deficits in the LH protocol. In turn, exposure to the LH protocol had significant effects on subsequent impulsive choice in the rGT. Second, these effects were opposite in the target LH group as compared to Learning controls. Finally, effects induced by the LH protocol were different in rats with high baseline impulsivity levels compared to those with low baseline impulsivity levels.

Baseline impulsivity levels did not correlate with subsequent escape deficits induced by the LH protocol. To the extent that such escape deficits are commonly regarded as an index of depressive-like failure to cope or ‘helplessness’ behaviour, these observations suggest that stable impulsivity traits may not in themselves be predictors or risk factors for depressive-type behaviour.

In contrast to the apparent lack of effect of basal impulsivity on LH-induced escape deficits, the effects of the LH protocol on subsequent impulsive choices in the rGT were pronounced. Rats in the target ‘helplessness’ group showed a small but significant increase in P4 responses compared to their own pre-LH levels, with correspondingly less responding for P3 and P1 options. In contrast, LC rats showed a significant increase in optimal P2 responding and a corresponding decrease in P4 responding, suggesting a clear improvement in decision-making behaviour in this group. Cage control rats showed an overall improvement in P2 responding at the expense of P1 and P3 responding while P4 responding was not altered, suggesting that this control group may have simply benefitted from additional trials.

The fact that opposite changes in P4 responding were observed in LH vs. LC rats (Fig. 5B and C) was unexpected and merits further consideration. One readily apparent difference between these groups is that LH rats were exposed to two stress sessions (one inescapable and one escapable) whereas the LC group was exposed to only one (escapable) stress session. It could thus be argued that the different effects of the LH protocol on subsequent rGT responding could have simply resulted from the additional stress experienced by the LH group compared to the LC group. This however seems unlikely for it would imply that behavioural effects on rGT responding in the LC group should be accentuated in the LH group as a result of additional stress. Instead, what was observed was a *decrease* in P4 responding in LC rats and an *increase* in P4 responding in LH rats.

An alternative and possibly more likely explanation would focus on the fact that the LH group had an experience of inescapable stress that subsequently compromised responses to escapable stress, whereas the LC group only experienced escapable stress. It is conceivable that the experience of escapable, and hence “controllable”, stress could account for the beneficial effects seen in the LC group. As noted below, if this suggestion is correct it would fall in line with other evidence suggesting that controllable stress may serve as “behavioural training” thereby exerting positive effects on behaviour^27^.

Further analysis revealed that baseline impulsivity in rGT, while not a predictor of depressive-type behaviour, interacted strongly with the LH protocol in affecting subsequent decision-making behaviour (Fig. 6). Thus, following exposure to the LH protocol choice of the riskier P4 option was significantly increased in High-impulsivity but not in Low-impulsivity rats. However this increase in P4 responding in High-impulsivity rats only occurred in rats in the LH group. Strikingly, High-impulsivity rats in the LC group actually *decreased* P4 responding on retest. These results appear to have uncovered an interesting role for baseline impulsivity interacting with stress to affect subsequent decision making behaviour.

### Stress effects on decision making

Learned helplessness involves exposure to inescapable stress. Previous animal studies have addressed general effects of stress on decision-making. Exposure to chronic unpredictable stress in adolescence was found to negatively impact aspects of decision-making^28^ and risk-assessment^29^, and to increase impulsive choice in adulthood. Rats receiving chronic corticosterone during adolescence (mimicking the effects of chronic stress) showed increased impulsive choice marked by an increased preference for small-immediate rewards rather than larger-delayed ones in a delayed-discounting task^30^. Effects of acute stress on decision-making have been less clear. Shafiei and co-workers reported that one hour of restraint stress did not alter preference between small/immediate *vs*. larger/delayed rewards in a delay-discounting task^31^. Koot *et al*.^11^ used a different adaptation of the Iowa Gambling Task for rats (rIGT) to assess the effects of corticosterone given 30 or 180 min prior to testing. Control rats improved performance on the rIGT over blocks of trials by decreasing the number of visits to the high-risk/disadvantageous arm of the apparatus, shifting choice preference toward the long-term advantageous arm. This however was not observed in rats given corticosterone 30 min prior to testing; in fact, the latter group consistently preferred the disadvantageous arm.

The present results are similar to those of Koot *et al*.^11^ using a different stressor and a different paradigm to assess decision-making. It thus seems possible to suggest that in our study the LH condition induced an apparent risk-proneness profile and impaired optimal use of choice strategy. This would be in line with a growing body of clinical evidence finding impulsive decision-making and increased risk-taking following stress exposure. Clinical studies have reported impulsive decision-making in healthy subjects submitted to acute stress^12^,^32^, in pathological gamblers^33^,^34^,^35^, and in depressed individuals^36^,^37^. It is unclear exactly how stress impacts subsequent decision-making. It is known, however, that key brain regions implicated in decision-making overlap to some extent with those that are involved in stress reactions and are therefore sensitive to stress-induced changes.

Chronic stress in rodents has been reported to disrupt decision-making by impairing the ability to select appropriate actions on the basis of previously-learned outcomes and the ability to shift to goal-directed behaviour. In particular, animals exposed to chronic stress show an impaired ability to perform based on the consequences of their previous choices^38^. It is also possible that stress effects on reward-related processing may play a role in the observed decision-making changes. Stress enhances sensitivity to appetitive behaviours such as drug or food intake^39^,^40^,^41^ and gambling^42^. Corticosteroids released during stressful situations have been linked to altered dopamine activity in the nucleus accumbens^43^,^44^,^45^ and prefrontal cortex, both of which are regions implicated in incentive reward processing and motivated behaviours^46^,^47^. For example, acute footshock increases dopamine release in these brain areas^48^ and increased in dopaminergic signalling in the striatum in response to acute stress has been linked to increased reward-seeking in rodents^15^,^49^,^50^,^51^,^52^. It is thus conceivable that stress-induced modulation of these brain regions may increase sensitivity to reward, promote risk-taking, and increase preference for impulsive options associated with immediate potential gains, regardless of negative long-term consequences.

### Could stress controllability be a factor?

Irrespective of specific mechanism(s) involved, a striking finding in the current study was the unexpected improvement in rGT decision-making in Learning control rats. It has been previously demonstrated that exposure to inescapable footshocks disrupts subsequent behavioural functions including avoidance learning, whereas exposure to identical but escapable shocks blunts such deficits^15^,^51^,^52^,^53^. Similar effects have been observed when animals are allowed some degree of control over stressful events^54^,^55^. Conceivably, prior exposure to escapable stress may help organisms develop more effective strategies to cope with future challenges such as decision-making by increasing vigilance or sensitivity to losses^56^. It seems therefore possible to suggest that the experience of stress controllability may at least in part account for the beneficial effects observed in the LC group, particularly in high-impulsivity rats. This suggestion could be tested using yoked control paradigms. If this hypothesis is confirmed it could provide a basis for potentially using behavioural training strategies in problem gambling in humans. Our results would further suggest that this might be particularly helpful in individuals with high baseline impulsivity levels.

In summary, while baseline impulsivity did not predict escape deficits in the LH paradigm, exposure to the LH protocol resulted in a subsequent increase in risky decision-making in the GT. This however occurred only in rats in the target LH group, for subsequent choice performance was actually improved in learning control rats. Further, these effects were primarily restricted to subjects with high baseline impulsivity levels in the rGT. The exact mechanism underlying these interactions between stress in the LH protocol and decision-making remains to be determined but the data are consistent with the possibility of opposite effects of controllable vs. uncontrollable stress on decision-making processes, a possibility that would be of translational interest and should thus be addressed in future work.

## Additional Information

**How to cite this article**: Nobrega, J. N. *et al*. Strong interactions between learned helplessness and risky decision-making in a rat gambling model. *Sci. Rep.* **6**, 37304; doi: 10.1038/srep37304 (2016).

**Publisher’s note**: Springer Nature remains neutral with regard to jurisdictional claims in published maps and institutional affiliations.

## Supplementary Material

Supplementary Material

## Figures and Tables

**Table 1:** Group sizes for experimental conditions^a^.

	Impulsivity			Total
	Low (LI)	Medium (MI)	High (HI)	
Cage Controls (CC)	2	4	2	8
Learning Controls (LC)	3	4	3	10
Learned Helplessness (LH)	10	7	10	27
Total	15	15	15	45

^a^See text for description of each experimental condition.

**Table 2:** Correlations between baseline rGT measures and avoidance latencies.

	N	Mean latencies		Number of trials having latencies of		
		All 15 trials	Last 3 trials	<5 sec	5–10 sec	>20 sec
**(I) Impulsivity score**^a^						
All rats	37	−0.19	−0.22	0.19	0.11	−0.20
LH group	27	−0.22	−0.27	0.24	0.12	−0.24
LC group	10	−0.23	−0.27	0.10	0.36	−0.20
**(II) Premature responding**						
All rats	37	−0.21	−0.17	0.25	0.03	−0.24
LH group	27	−0.07	0.09	−0.07	−0.09	0.09
LC group	10	−0.28	−0.10	0.32	0.09	−0.33

^a^Impulsivity score = (P4 + P3)/(P2 + P1).
