# Supplementary material for: Strong interactions between learned helplessness and risky decision-making in a rat gambling model
Source: Sci Rep. 2016 Nov 18;6:37304. doi: 10.1038/srep37304 (PMC5114549; doi:10.1038/srep37304)
Supplement: Supplementary Material [file srep37304-s1.doc]

**Supplementary Material**

***Scientific Reports***

***Revised MS SREP-16-18507C***

**Strong interactions between learned helplessness and risky decision-making in a rat gambling model**

José N. Nobrega ^*,2,3,4^, Parisa S. Hedayatmofidi ^*,1^, Daniela S. Lobo ^1,2,4^

**Suppl. Fig 1. Distribution of rGT responses prior to exposure to the Learned Helplessness protocol.** Prior to exposure to the Learned Helplessness protocol there were no significant differences among the three groups in the LH protocol.

**Suppl. Fig. 2. Distribution of rGT omissions, premature responses and perseverative responses before and after exposure to the learned helplessness protocol**. Premature responding was significantly reduced in the Cage Control group (N=8), *p* < 0.01). Perseverative responses were reduced equally in all groups on retest (*p* < 0.01).
